# Supplementary material for: Rapid turnover of life-cycle-related genes in the brown algae
Source: Genome Biol. 2019 Feb 14;20:35. doi: 10.1186/s13059-019-1630-6 (PMC6374913; doi:10.1186/s13059-019-1630-6)
Supplement: Supplementary file 3 — Protein alignments, corresponding Gblocks html file and CDS sequences for the lineage conserved gametophyte-biased genes. (ZIP 2109 kb) [file 13059_2019_1630_MOESM3_ESM.zip › Con_GA_alignments/Ec-05_002090.aa_ali.fasta-gb.htm]

Ec-05\_002090.aa.fasta.trans.aa\_ali.fasta


## Gblocks 0.91b Results

Processed file: **Ec-05\_002090.aa.fasta.trans.aa\_ali.fasta**  
Number of sequences: **2**  
Alignment assumed to be: **Protein**  
New number of positions: **204** (selected positions are underlined in blue)

```
                         10        20        30        40        50        60
                 =========+=========+=========+=========+=========+=========+
Ec-05_002090     MSESDGGSGPQHGGGGRKRANSSSTGGRNSPSPPRNASVSSASGASVTPAPSPAPAPAPG
MSTRG.23503.1    ------------------------------------------------------------
                                                                             


                         70        80        90       100       110       120
                 =========+=========+=========+=========+=========+=========+
Ec-05_002090     PTSKKKQKKGSRGSGTGSQAYIPPGVVISRSAEDCAPQLTMPHKDQLTVTGHKGFRMARA
MSTRG.23503.1    -------------------------------------------QDQLTVVGNKGFRMARA
                                                             ################


                        130       140       150       160       170       180
                 =========+=========+=========+=========+=========+=========+
Ec-05_002090     TKGVSNGTWYWECAVLPPETTEGHCRLGWSLPAGKLQGPVGYDKFSYAYRDIAGSKVHDS
MSTRG.23503.1    THGVSNGTWYWECTVLPPETKEGHCRLGWSLATGSLQAPVGYDKSSYAYRDIAGSKVHES
                 ############################################################


                        190       200       210       220       230       240
                 =========+=========+=========+=========+=========+=========+
Ec-05_002090     HRSDNYGEPWGPGDVIGFLIRLKSPTPEEIIDTVGASSDSGRHGMTAAAAAAALAAGQSP
MSTRG.23503.1    LRTDNYGEPWGQGDVIGFLIKLKSPSEEELKDTVGAASDSGR--LSVAAAAAALAAGQSP
                 ##########################################     #############


                        250       260       270       280       290       300
                 =========+=========+=========+=========+=========+=========+
Ec-05_002090     SELAATAASDTAAG--DPLNEIRFFKNGRDQGVAYSAMKPG--------------ISRLG
MSTRG.23503.1    SERAAKAAEAVAAGGANAFNEIRFFKNGRDQGIAFSGIKPGTYFPAVSLYMGGSVRANFG
                 ##############     ######################                  #


                        310       320       330       340       350       360
                 =========+=========+=========+=========+=========+=========+
Ec-05_002090     AVAVHPLLKLEGGSSVAPAG--------CKRPPHADHYVSKSQEFVVCL-----------
MSTRG.23503.1    PEFVYPPPRSYGWKPVAALRPFTKDEIKSQRNTIKRERASRGLETATRLIGGAPPLPSLN
                 #################             ###################           


                        370       380       390       400       410       420
                 =========+=========+=========+=========+=========+=========+
Ec-05_002090     ------------------------------------------------------------
MSTRG.23503.1    GSGNTSGNGSGSSRSASPLPPPSVGPSKNDKTLDKGKGSRGEGEGSGKGRASPPPKDDEE
                                                                             


                        430
                 =========+
Ec-05_002090     ---------N
MSTRG.23503.1    GEKRSGGGAA
```

```
Parameters used
Minimum Number Of Sequences For A Conserved Position: 2
Minimum Number Of Sequences For A Flanking Position: 2
Maximum Number Of Contiguous Nonconserved Positions: 8
Minimum Length Of A Block: 10
Allowed Gap Positions: None
Use Similarity Matrices: Yes
```

```
Flank positions of the 5 selected block(s)
Flanks: [105  222]  [228  254]  [260  281]  [300  317]  [331  349]  

New number of positions in Ec-05_002090.aa.fasta.trans.aa_ali.fasta-gb:  204  (47% of the original 430 positions)
```
